# Multi-omics analysis reveals the interplay between pulmonary microbiome and host in immunocompromised patients with sepsis-induced acute lung injury

**DOI:** 10.1128/spectrum.01424-24

**Published:** 2024-10-18

**Authors:** Fan Lu, Ting Huang, Ruichang Chen, Haiyan Yin

**Affiliations:** 1 Department of Emergency, The First Affiliated Hospital of Jinan University, Guangzhou, China; 2 Department of Obstetrics, Guangdong Women and Children Hospital, Guangzhou, China; 3 Intensive Care Unit, The First Affiliated Hospital of Jinan University, Guangzhou, China; Agroscope, Nyon, Switzerland

**Keywords:** sepsis, pulmonary microbiome, metagenomic next-generation sequencing

## Abstract

The mechanisms behind the high inflammatory state and immunocompromise in severe sepsis remain unclear. While microbiota’s role in immune regulation is known, the impact of pulmonary microbiota on sepsis progression is not fully understood. This study aims to investigate pulmonary microbial characteristics in septic patients and their relationship with host immune-related genes and clinical features. Fifty-four sepsis patients were divided into the immunocompromised host (ICH) group (*n* = 18) and the control group (*n* = 36). Bronchoalveolar lavage fluid (BALF) was analyzed using metagenomic next-generation sequencing (mNGS) to assess the pulmonary microbiome, and transcriptomic sequencing evaluated host gene expression. The pulmonary microbiota network in the ICH group showed notable alterations. Symbiotic bacteria like *Streptococcus salivarius* and *Streptococcus oralis* were key taxa in the control group. In contrast, opportunistic pathogens such as *Campylobacter concisus* and *Prevotella melaninogenica*, typically linked to infections in various body sites, dominated in the ICH group. Transcriptomic analysis revealed differential genes between the two groups. The downregulated differential genes in the ICH group were primarily enriched in pathways related to T-cell activation and the Type I interferon signaling pathway, both crucial for the immune system. Further correlation analysis identified significant associations between certain microbes and host genes, as well as clinical indicators, particularly with species like *Campylobacter concisus*, *Streptococcus salivarius*, *Streptococcus oralis*, and several species of *Veillonella*. These findings suggest that alterations in the pulmonary microbiome, especially the presence of opportunistic pathogens, may contribute to immune dysregulation in immunocompromised septic patients, warranting further research to explore causal relationships.

**IMPORTANCE:**

Recent research has substantiated the significant role of microbiota in immune regulation, which could influence high inflammatory state and immunocompromise in patients with severe sepsis, as well as provide new opportunities for acute lung injury induced by sepsis diagnosis and treatment. Our study identified some potential critical microbes (Campylobacter concisus and several species of Veillonella), which were correlated with immune-related genes and might be the novel target to regulate immunotherapy in sepsis.

## INTRODUCTION

Sepsis, a common infectious disease triggered by the invasion of pathogenic microorganisms, often presents with systemic inflammatory response syndrome and multi-organ dysfunction. This condition not only leads to substantial hospitalization costs but also has a high in-hospital mortality rate (1). Notably, the lung is a primary site of infection in sepsis, which can further lead to acute lung injury (ALI) or acute respiratory distress syndrome (ARDS) (2). Although the mortality rate of sepsis has significantly decreased over the past few decades, thanks to the use of various antibiotics, timely fluid resuscitation, and organ support therapies, the concurrent or sequential occurrence of immune suppression and inflammation remains a significant challenge in treatment (3, 4). Currently, immunosuppression is increasingly recognized as one of the main causes of death in sepsis (5, 6). Specifically, the immunosuppression induced by sepsis usually results from the disruption of immune homeostasis, characterized by aberrant death of immune system effector cells, release of anti-inflammatory cytokines, expression of immune checkpoint, and over-proliferation of immune suppressor cells (7). Recent randomized clinical trials have demonstrated that immunomodulatory therapy can improve organ function and reduce mortality in patients with severe sepsis (8). Therefore, a deeper investigation and understanding of the immunosuppression mechanisms triggered by sepsis are crucial for developing new therapeutic strategies and improving clinical outcomes for patients.

As microbes and hosts have co-evolved in a symbiotic relationship, the microbiota plays a crucial role in regulating immune system functions. It includes influencing the differentiation and activation of T cells, antibody production, phagocytic activity of macrophages, and local barrier functions (9, 10). The gut microbiota, one of the most extensively studied microbial communities, plays a pivotal role in the immune system. Dysbiosis of gut microbiota can lead to immune dysregulation, contributing to the development of intestinal and systemic diseases (11). Compared to the gut microbiota, the function and impact of the lung microbiome require more extensive research. Current studies suggest the existence of a dynamic microbial equilibrium in healthy lungs (12). Changes in lung structure and the lung microenvironment (such as mucosal pH, alveolar-arterial oxygen gradient, nutrients, and temperature) can alter the composition of the lung microbiome (13). Limited research had indicated that certain organ dysfunctions associated with sepsis, such as ALI, acute kidney dysfunction, and encephalopathy, are related to microbial imbalance (10). In patients with sepsis and ARDS, alterations in the lung microbiome have been observed, including the enrichment of gut-specific bacteria (14). The lung microbiome, by modulating host immune responses and inflammation, may contribute to the pathogenesis of pulmonary diseases and affect critical pathophysiological processes, including epithelial cell apoptosis, collagen deposition, oxidative stress, and airway remodeling (15). However, how the lung microbiome composition changes in immunocompromised patients related to sepsis and whether these changes impact the host’s immune status remain unclear.

In this study, we aim to characterize the pulmonary microbial community in patients with sepsis-induced immunocompromised states and investigate the relationship between the pulmonary microbiota and the host’s immune-related genes and clinical features in these patients. These comprehensive analyses are expected to enhance our understanding of the role of pulmonary microbiota in the progression of sepsis.

## RESULTS

### Demographic and clinical characteristics of participants

**Fig 1:**
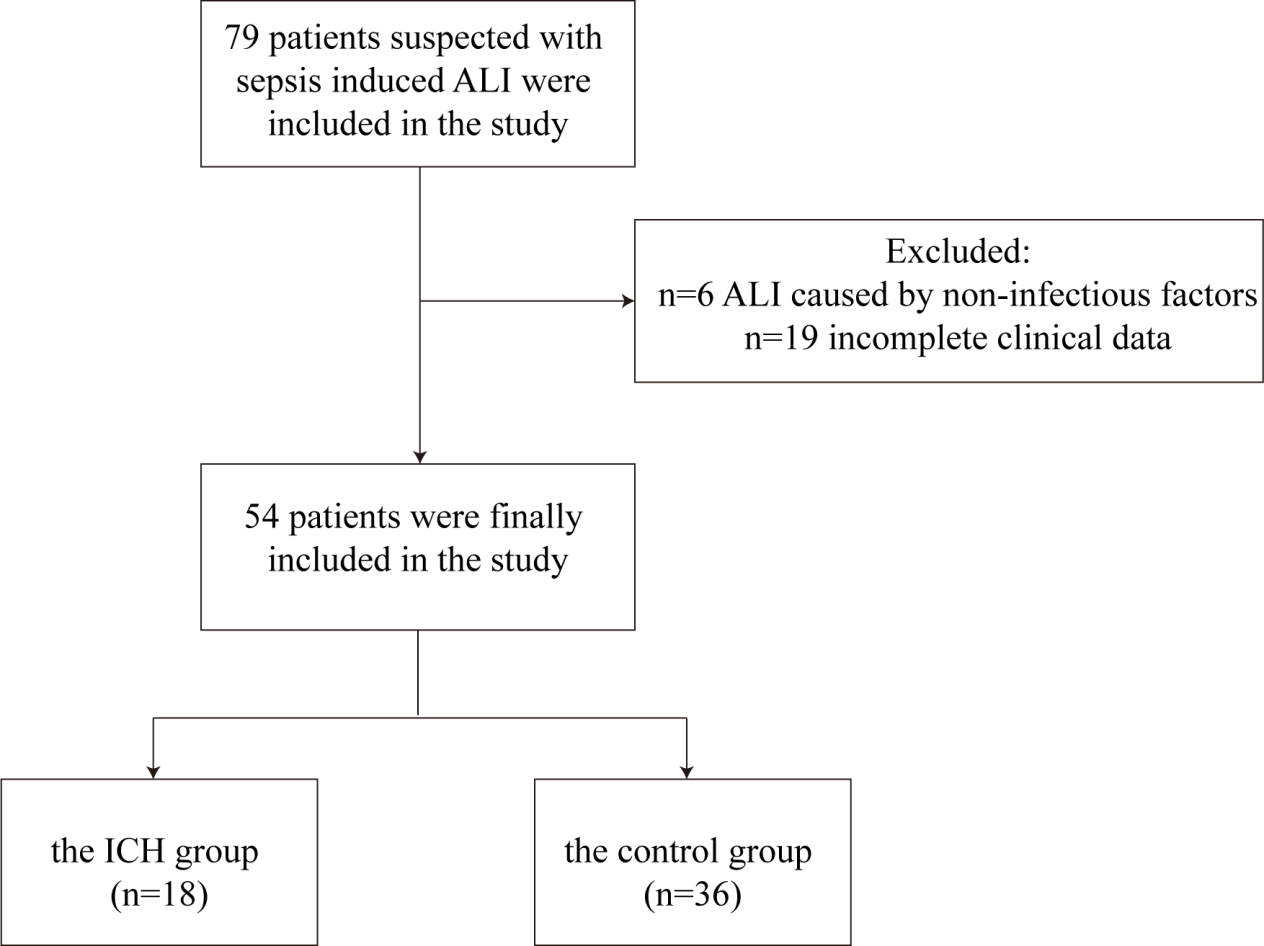
The flowchart of the recruitment process for study participants.

The study initially included 79 patients with sepsis-induced ALI. Of these, 19 patients had incomplete medical records, and 6 patients had ALI due to non-infectious factors. Ultimately, 54 patients were included in the final analysis. Based on whether they had compromised immune function, the patients were divided into the ICH group (*n* = 18) and the control group (*n* = 36). The flowchart of the recruitment process for study participants is shown in Fig. 1. In the ICH group, the levels of hemoglobin were significantly lower compared to the control group, with the observed differences being statistically significant (84.078 ± 28.528 g/L vs 101.747 ± 30.492 g/L, *t*-test, *P* = 0.04, Table 1). However, no significant differences were identified between the two groups in other variables, including age, gender, length of ICU stay, and biochemical parameters, such as white blood cell counts (*P* > 0.05, Table 1).

**TABLE 1:** Demographic and clinical characteristics of participants^*a*^

	ICH group (*n* = 18）	Control group (*n* = 36）	*P* value
Men (%)	15 (83.3%)	26 (72.2%)	0.57
Age (year)	65.94 ± 13.93	61.58 ± 16.27	0.31
Total length of hospital stay (day)	30.00 (13.25, 37)	16.50 (10.00, 27.00)	0.09
Length of ICU stay (day)	15.00 (6.00, 21.75)	10.50 (8.00, 22.00)	0.81
SOFA score	10.280 ± 3.770	9.194 ± 3.875	0.33
APACHII score	29.00 (23.25, 33.75)	28.00 (22.00, 34.25)	0.67
WBC (×10^9^ /L)	12.39 (7.47, 20.05)	14.28 (10.56, 10.44)	0.47
ANC (×10^9^ /L)	10.95 (6.37, 18.54)	12.60 (9.15, 18.56)	0.51
CRP (mg/L)	116.27 (81.68, 194.85)	108.68 (26.75, 178.27)	0.29
PCT (ng/L)	5.101 (1.462, 37.972)	1.780 (0.662, 7.841)	0.07
NEUT (%)	86.35 (79.83, 89.58)	89.54 (85.35, 92.53)	0.15
HB (g/L)	84.078 ± 28.528	101.747 ± 30.492	0.04
PLT (g/L)	146.494 ± 110.544	192.483 ± 122.206	0.19
Lactic acid (mmol/L)	3.722 ± 3.713	4.914 ± 6.333	0.47
pH	7.333 ± 0.176	7.400 ± 0.116	0.10

^*a*^ WBC, white blood cell; ANC, absolute neutrophil count; CRP, C-reactive protein; HB, hemoglobin; PLT, platelet; pH, potential of hydrogen.

### Pulmonary microbiota characteristics of participants

**Fig 2:**
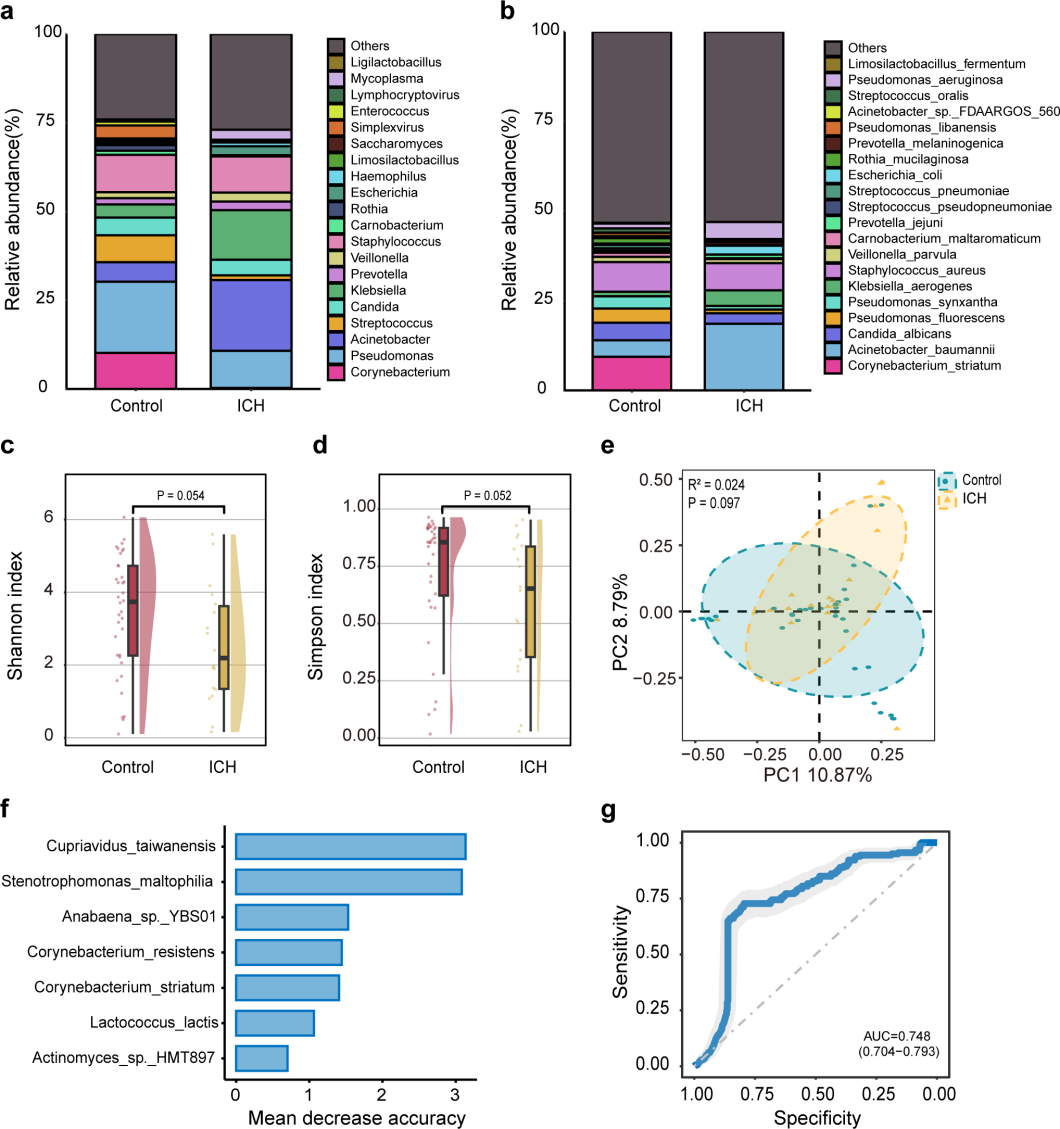
Pulmonary microbiota characteristics of participants. (a) The top 20 most abundant genera of pulmonary microbial communities in the ICH group and the control group. (b) The top 20 most abundant species of pulmonary microbial communities in the ICH group and the control group. Comparison of the (c) Shannon index and (d) Simpson index between the immunocompromised group and control group. The Shannon and Simpson indices between the two groups were compared using the Wilcoxon rank sum test. (e) PCA analysis based on Bray–Curtis distance between the two groups. PERMANOVA test was used to compare the difference between beta diversity among the two groups. (f) The seven microbes with the most weight to discriminate immunocompromised hosts and controls were selected by the random forest models. The length of the bar indicated the importance of the microbes for classification. (g) Receiver operating characteristic analysis was performed to evaluate the diagnostic performance of these seven microbial biomarkers.

To assess the characteristic of lung microbiota between the two groups, we analyzed the top 20 most abundant genera and species. At the genus level, *Acinetobacter* (19.94%) and *Klebsiella* (13.98%) were the predominant genera in the ICH group (Fig. 2a). The control group exhibited a dominance of *Pseudomonas* (20.03%) and *Corynebacterium* (10.17%) as the leading genera. At the species level, the main species in the control group were *Corynebacterium striatum* (9.36%), *Staphylococcus aureus* (8.28%), and *Candida albicans* (4.91%), while the main species in the ICH group are *Acinetobacter baumannii* (18.60%), *S. aureus* (7.60%), and *Pseudomonas aeruginosa* (4.72%) (Fig. 2b).

Subsequently, we conducted a further analysis of the diversity of the pulmonary microbiota. Shannon and Simpson indices, which reflect the richness and evenness of the microbial community, showed no significant differences in the lung microbiota between the two groups (Shannon index, Wilcoxon rank sum-test, *W* = 429, *P* = 0.054, Simpson index, Wilcoxon rank sum test, *W* = 430, *P* = 0.052, Fig. 2c and d). This suggests a similar level of alpha diversity in the pulmonary microbiota. Additionally, a PCA analysis based on the Bray-Curtis distance revealed that the samples from the two groups largely overlapped (Fig. 2e), indicating no substantial difference in the beta diversity of the pulmonary microbiota between these groups. To further assess this, we performed a PERMANOVA (Permutational Multivariate Analysis of Variance) test, which confirmed that there was no significant difference between the groups (PERMANOVA, *P* = 0.097, *R*^2^ = 0.024).

We then identified the most discriminative biomarkers by constructing a random forest model. *Cupriavidus taiwanensis*, *Stenotrophomonas maltophilia*, *Anabaena sp YBS01*, *Corynebacterium resistens*, *C. striatum*, *Lactococcus lactis*, and *Actinomyces sp HMT897* were identified as crucial microbes for distinguishing between the two groups (Fig. 2f). The random forest model yielded an AUC value of 0.748, indicating a strong discriminatory power within this classifier (Fig. 2g).

### Co-occurrence network patterns of pulmonary microbiome

**Fig 3:**
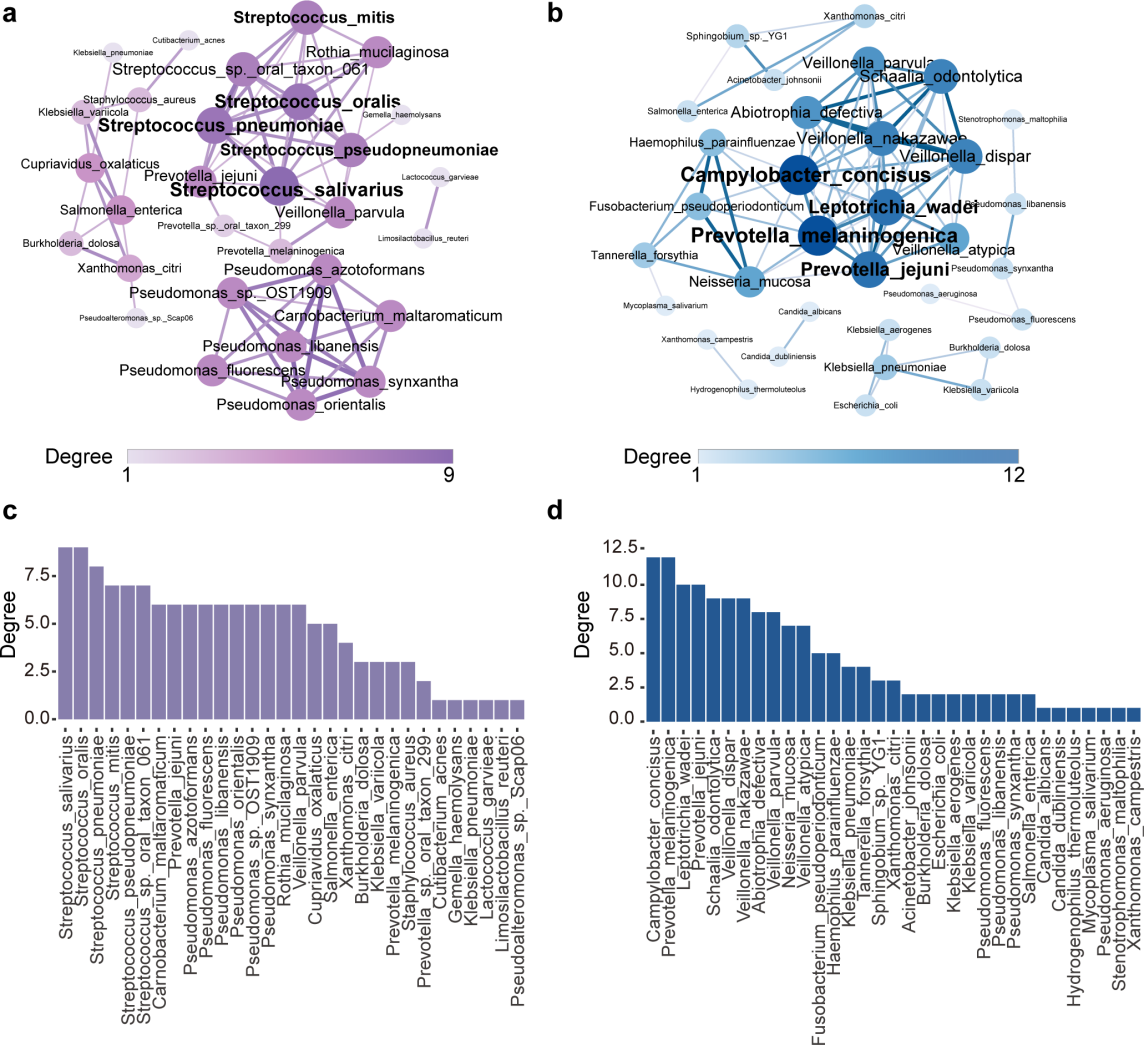
Co-occurrence network pattern of pulmonary microbiome in the control group and ICH group. (a) Pulmonary microbial network of the control group. (b) Pulmonary microbial network of the ICH group. Nodes with a correlation coefficient greater than 0.6 and a *P* value less than 0.05 are retained in the network diagram. The size and color of nodes represent the degree in the network diagram. The width of the edge represents the magnitude of the correlation coefficient. (c) The degree of each node in the microbial network of the control group. (d) The degree of each node in the microbial network of the ICH group.

Microbes do not exist independently in the ecological environment, and they are mutually beneficial or mutually exclusive (16). Therefore, we selected the highest abundance of 100 microbes at the species level in two groups to construct a microbial co-occurrence network diagram (Fig. 3a and b). In this network, the degree of node refers to the number of edges associated with it. Some scholars posited that nodes with the highest degree in a network might be considered keystone taxa, potentially playing significant roles within that network (17). Consequently, we calculated the degree for each bacterial species in the network to identify core species within the microbial community (Fig. 3c and d). In the control group, *Streptococcus salivarius* and *Streptococcus oralis* had the highest degree. These two *Streptococcus* species are recognized as early colonizers of the upper respiratory mucosa. Their numerical predominance is generally indicative of a relatively healthy microbial community (18). In the ICH group, *Campylobacter concisus* and *Prevotella melaninogenica* had the highest degree. These two bacterial species have been reported in the context of inflammation or infectious diseases, with the latter frequently observed in patients with immunodeficiency or those experiencing surgical complications (19–21). *Veillonella dispar*, *Veillonella nakazawae*, *Veillonella parvula*, and *Veillonella atypica* also exhibit a higher degree. This suggests that in sepsis patients experiencing immune suppression, there are significant changes in the pulmonary microbial network. Notably, potential pathogens have emerged as keystone species within this network.

### Host transcriptome analysis of participants

**Fig 4:**
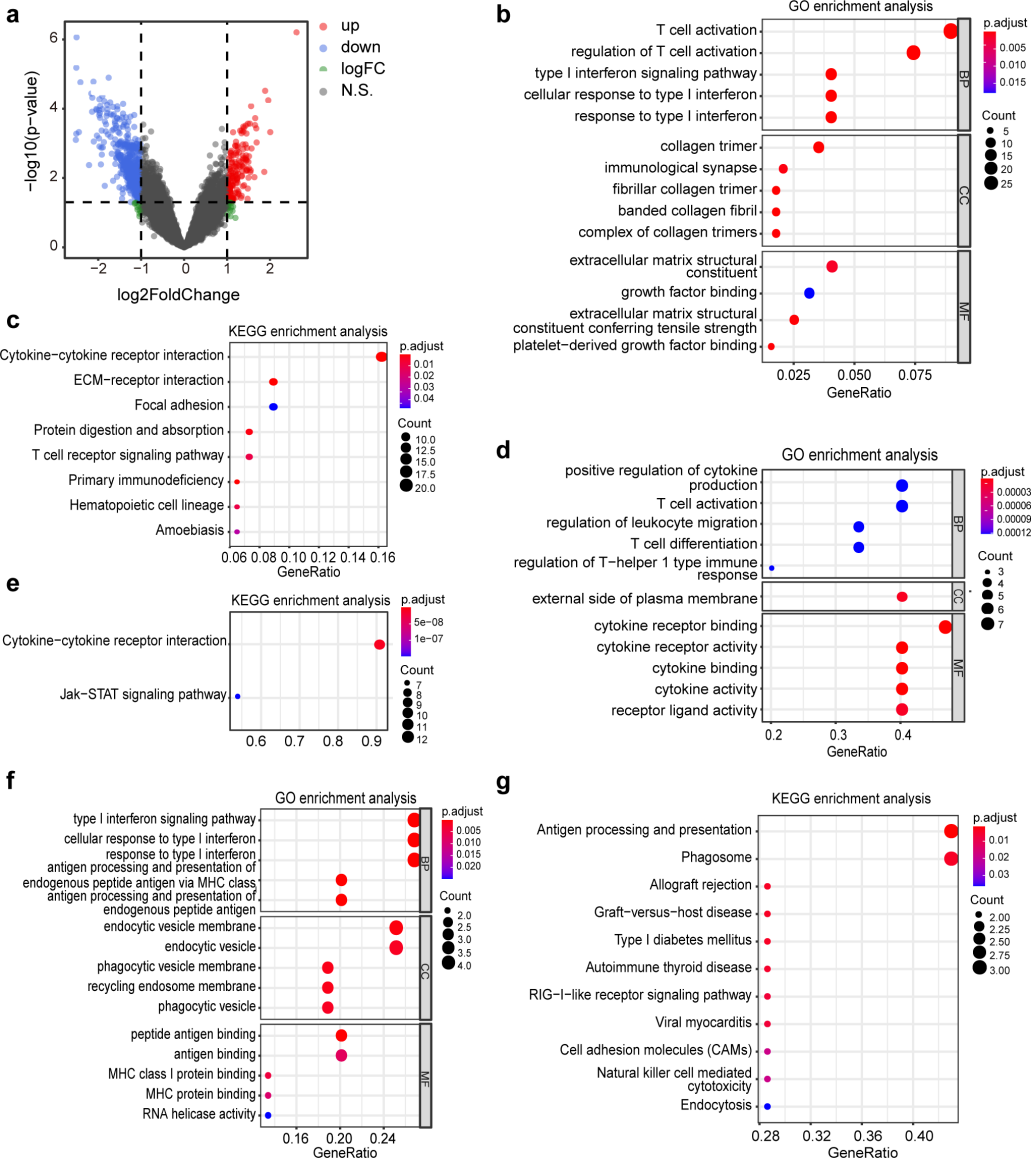
Results of transcriptomic analysis in the ICH group and the control group. (a) Volcano plot of gene expression between two groups. Each point represents a gene. Significantly upregulated genes, significantly downregulated genes, and genes with non-significant expression differences are marked with different colors. (b) GO enrichment pathway analysis of downregulated differentially expressed genes in the ICH group vs control group. GO database classification includes biological process (BP), cellular component (CC), and molecular function (MF). The *x*-axis represents the ratio of differentially expressed genes annotated to GO Terms to the total number of differentially expressed genes. The *y*-axis represents the GO Terms. The size of the dots indicates the number of genes annotated to each GO Term. The color gradient from red to blue represents the significance of enrichment, with redder colors indicating greater significance. (c) KEGG enrichment pathway analysis of downregulated differentially expressed genes in the ICH group vs control group. (d) GO enrichment pathway analysis of significantly differentiated immune-related genes. (e) KEGG enrichment pathway analysis of differentiated immune-related genes. (f) GO enrichment pathway analysis of significantly differentiated genes related to interferon induction. (g) KEGG enrichment pathway analysis of differentiated genes related to interferon induction.

The transcriptomic analysis provides a comprehensive reflection of the expression patterns of all RNA molecules (22). It offers invaluable insights into the global gene activity under particular environmental conditions, or during distinct physiological and pathological states (23). Therefore, to evaluate the gene expression profiles of sepsis-induced ALI patients under different immune states, we conducted transcriptome sequencing on the BALF of the subjects. In total, 672 DEGs were identified, of which 164 genes were upregulated and 508 genes were downregulated in the ICH group (Fig. 4a). Subsequently, we ascertained the primary biological functions exerted by the DEGs through GO functional significance enrichment analysis. The results of GO enrichment analysis indicated that the most significantly different pathways between the two groups were the regulation of T cell activation and the type I interferon signaling pathway (Fig. 4b). The KEGG database amalgamates genomic, chemical, and systemic functional information. Therefore, we conducted a KEGG pathway enrichment analysis on the differential gene sets. The KEGG pathway enrichment analysis revealed that the most significantly different pathways between the two groups were cytokine-cytokine receptor interaction, ECM-receptor interaction, primary immunodeficiency, and the T cell receptor signaling pathway (Fig. 4c). These results corroborated the presence of differential immune function between the subjects.

Given the varying immune statuses of our study subjects, we focused our attention on genes related to immunity and interferon induction. We identified 255 immune-related genes utilizing the ImmPortDB database, among which 15 exhibited intergroup differences (Fig. S1). GO enrichment pathway analysis indicated that the differentially expressed immune-related genes between the two groups were primarily enriched in pathways related to cytokine and receptor activity, cytokine and receptor binding, as well as T cell activation and differentiation pathways (Fig. 4d). The KEGG pathway analysis results suggested that the differentially expressed immune-related genes between the two groups were enriched in the cytokine-cytokine receptor interaction and the Jak-STAT signaling pathway (Fig. 4e). Additionally, we identified 380 genes associated with interferon induction, among which 16 exhibited intergroup differences (Fig. S2). The GO enrichment pathway analysis indicated an enrichment of interferon induction-related differentially expressed genes primarily in pathways such as the type I interferon signaling pathway, response to type I interferon, and antigen processing and presentation of endogenous peptide antigen via MHC class I molecules (Fig. 4f). Meanwhile, the KEGG enrichment analysis indicated that the differentially expressed genes related to interferon induction were predominantly concentrated in antigen processing and presentation, and phagosome pathways (Fig. 4g). Therefore, the immunosuppression of sepsis-induced ALI may be related to immune cell-mediated immune responses, interferon signaling pathways, and MHC. The activation of T cells and the response to type I interferon may play an important role in this process.

### Associations between microbiota, genes, and clinical data

**Fig 5:**
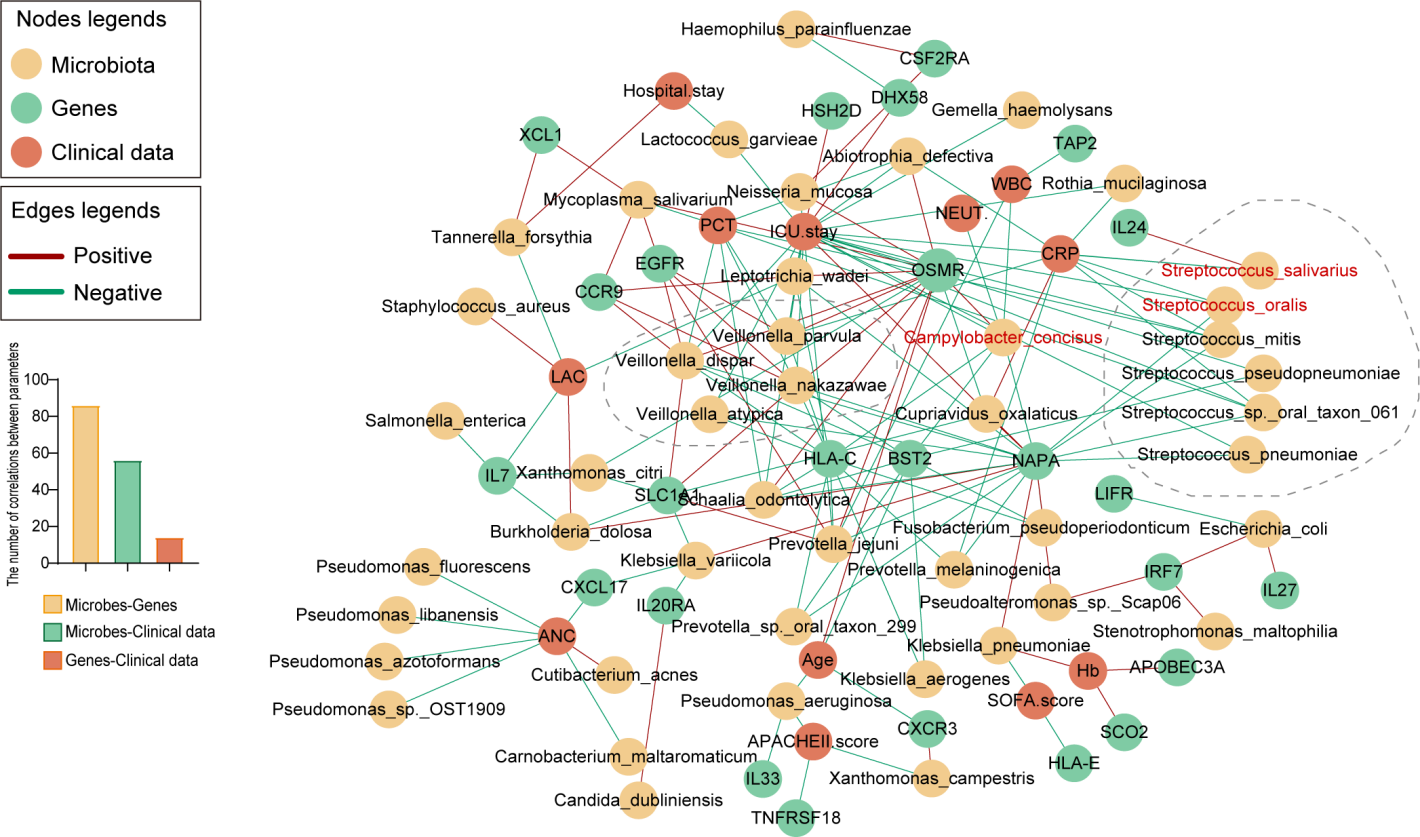
Associations of microbes, genes, and clinical data. Yellow nodes represent microbes, green nodes represent genes, and red nodes represent clinical data. Red edges indicate a positive correlation between nodes, while green edges indicate a negative correlation between nodes.

To investigate the relationships among pulmonary microbiota, immune and interferon-related genes, and clinical indicators, we constructed a correlation network to illustrate the interactions between these three categories of variables (Fig. 5). Several species of *Streptococcus*, including keystone species in the microbial network of the control group such as *S. oralis* and *S. salivarius*, exhibited a negative correlation with patient’s CRP levels and OSMR expression. It is reported that the OSMR gene is significantly elevated in septic myocarditis cell models (24), suggesting its potential role in the progression of sepsis. This implies that the aforementioned *Streptococcus* species may exert a beneficial effect by reducing CRP and OSMR levels. *C. concisus*, a keystone species in the microbial network of the immunocompromised group, exhibited a negative correlation with white blood cell count and neutrophil percentage, and a positive correlation with OSMR. It indicated that *C. concisus* influences inflammatory responses and immune regulation by affecting the expression of the OSMR gene. Furthermore, species of *Veillonella* (*V. atypica*, *V. dispar*, *V. nakazawae*, *V. parvula*) in the immunocompromised microbial network showed positive correlations with OSMR and EGFR, but negative correlations with HLA-C and BST2. Additionally, our research indicates that the number of correlations between microbiota and host genes is greater than that between microbiota and clinical indicators. This finding may suggest a more intricate interaction between the microbiome and the host’s genetic background.

## DISCUSSION

In this study, we evaluated the characteristics of pulmonary microbiota in patients with sepsis-associated ALI, both immunocompromised and non-immunocompromised, and examined the correlation between the microbiota and host gene expression as well as clinical features. In patients with sepsis-associated ALI who are immunocompromised, a significant alteration in the pulmonary microbiota network pattern was observed, along with a notable downregulation of T-cell activation, regulation, and interferon signaling pathways. The connection between the pulmonary microbiota and host gene expression was found to be more pronounced than its correlation with clinical indices, suggesting that the pulmonary microbiota could influence host phenotypes through modulation of gene expression. This study provides a novel perspective on the changes in the pulmonary microbiome in immunocompromised patients with sepsis-induced ALI. The strong link between pulmonary microbiota and host gene expression implies that the microbiota could be a potential modulator influencing the disease progression and prognosis.

The characteristics of the pulmonary microbiota in patients with sepsis-induced ALI, particularly those with immunocompromised, have not been comprehensively reported to data. In our study, we identified *Acinetobacter* and *Klebsiella* as the dominant genera in the pulmonary microbiome of the ICH group. Notably, *Klebsiella*, typically a resident of the gut (25), when detected in the lungs, may indicate a compromised intestinal barrier in sepsis patients. This breach potentially allows gut bacteria to enter the bloodstream and reach the lungs, thereby affecting pulmonary immune responses. Additionally, the concept of the gut-lung axis, highlighting the complex interactions between the gut and respiratory tract microbiota, has been proposed in academia. Changes in the gut microbiota are believed to impact lung health and vice versa. The gut-lung axis plays a pivotal role in diseases such as ALI (26), bronchiectasis (27), and chronic obstructive pulmonary disease (28). Regrettably, our study did not investigate the gut microbiota of the subjects, which represents a potential direction for future research. Moreover, although our study revealed no significant differences in the diversity of the pulmonary microbiota between the two groups, their microbial network patterns were distinctly different. In the control group, *S. salivarius* and *S. oralis* emerged as keystone species of the microbial network. These streptococci, typically found in the human oral cavity and respiratory tract, with *S. oralis* being a commensal and *S. salivarius* considered a probiotic, may aid in preventing or alleviating inflammation (29, 30). This suggests that in individuals who are not immunocompromised, the lung microbiota is predominantly composed of commensal or beneficial bacteria. In contrast, in the microbiota networks of immunocompromised individuals, *C. concisus* and *P. melaninogenica* were identified as keystone species. *C. concisus* is known as a gastrointestinal pathogen, but its role in the lungs remains unclear. *P. melaninogenica*, recognized as a periodontal pathogen in the oral cavity, is linked to a range of oral diseases, including periodontitis and oral lichen planus (31, 32). Intriguingly, this bacterium has also been identified in the extra-oral organs and tissues of patients suffering from systemic conditions such as ankylosing spondylitis and non-alcoholic fatty liver disease (33, 34). These findings indicate that ectopic colonization by *P. melaninogenica* may play a contributory role in the pathological mechanisms underlying these diseases.

By further transcriptome analysis, we found that expressions of the host genes had significant differences in two groups. The genes associated with cytokine cytokine receptor interaction significantly downregulated in ICH group, especially the part of T cell-mediated immune responses. Increasing evidence has also shown that T cells play a critical role in the immune response during sepsis, depletion, or exhaustion of T cells increased susceptibility to opportunistic nosocomial infection (35, 36). In addition, the DEGs in two groups were significantly enriched in the type I interferon signaling pathway and major histocompatibility complex (MHC). MHC class I regulates the number and functional responses of CD8^+^ T cells *in vivo* during sepsis, as well as antigen-specific CD8^+^ T cell proliferation *in vitro*, while CD8^+^ T cell numbers and functional responses are associated with host immune and clinical events in sepsis (37). IFN-β is reported to improve 28-day survival by preventing sepsis-induced immunosuppression in patients with ALI in a previous study (38). *In vitro* research, IFN-β improved bacterial clearance and alveolar neutrophil recruitment, restored alveolar innate immune responsiveness and reduced the odds ratio for 7-day mortality (39).

Microorganisms may influence host phenotypes by modulating the expression of host genes (40, 41). Accordingly, we analyzed the association between the lung microbiota, genetic expressions, and clinical indicators in subjects. In microbial networks, keystone species refer to those crucial for maintaining the structure and function of the entire system, exerting an influence on the ecosystem far beyond their relative abundance. Our focus was, thus, on the relationship between keystone species, gene expression, and clinical indicators. Intriguingly, we observed a negative correlation between OSMR and keystone species in the control group (*S. oralis* and *S. salivarius*), and a positive correlation with keystone species in the ICH group (*C. concisus*) and several *Veillonella* species. Oncostatin-M (OSM), a multifunctional cytokine belonging to the IL-6 family, primarily produced by monocytes and macrophages, exhibits both anti-inflammatory and pro-inflammatory properties depending on the cellular context (42). The signaling function of OSM is predominantly regulated by two distinct receptor complexes, OSMR I and OSMR II. OSMR is widely expressed on various tumor cells, epithelial cells, and endothelial cells. Current research on OSM predominantly focuses on its role in tumors and chronic inflammatory diseases. It has been reported that OSM expression correlates with the malignancy of gliomas, underscoring its association with lower survival rates in patients (43). Recent studies have found elevated levels of OSM and OSMR in conditions such as sepsis, septic shock, and septic myocarditis (24, 44). *S. oralis* and *S. salivarius*, as commensal bacteria of the host, exhibited a negative correlation with OSMR, potentially revealing their role in maintaining host immune stability. *C. concisus* is a potential pathogen. An increase in *Veillonella* abundance has been associated with inflammatory bowel diseases and autoimmune hepatitis (45, 46). Their positive correlation with OSMR may suggest a potential regulatory effect of these microbes on the host immune system during disease states. However, many unknowns remain regarding the specific functions and mechanisms of action of OSM and OSMR, particularly concerning how microbes may regulate OSMR gene expression. This opens new directions for future research in the field of microbe-host interactions. Furthermore, *Veillonella* also showed a positive correlation with EGFR and a negative correlation with HLA-C and BST2. EGFR, through the TBK1/Glut1 pathway, may lead to immune cell exhaustion in septic patients, potentially affecting the immune functional status (47). HLA-C alleles could increase the risk of infection and lower the risk of autoimmune disease by reducing immune reactivity to specific ligands (48). Interferon-induced bone marrow stromal cell antigen 2 (BST2) inhibits viral replication through an NF-κB-dependent pathway (49). We speculate that *Veillonella* might induce immunosuppression by promoting the expression of the EGFR gene and inhibiting the expression of BST2 and HLA-C genes. This hypothesis warrants further investigation and validation in future studies.

While this study provided insightful observations into the relationship between pulmonary microbiota and host gene expression in patients with sepsis-induced ALI, it is important to acknowledge certain limitations. First, the relatively small sample size may limit the generalizability of our findings. The complex nature of sepsis-associated acute lung injury implies that a larger cohort would be beneficial to validate our results and to ensure that they are representative of the broader patient population. This would enhance the statistical power of the study and potentially reveal more nuanced interactions between the microbiota and host responses. Furthermore, as a clinical observational study, our research does not establish a causal relationship between alterations in the pulmonary microbiota and changes in host gene expression. While we have identified significant correlations, these do not necessarily imply causation. The observed associations might be influenced by environmental factors or other confounding variables that were not controlled for in this study. Future research employing experimental models or longitudinal studies could help in elucidating the causal mechanisms underlying these associations.

### Conclusion

In this study, significant alterations were observed in the pulmonary microbial network patterns of patients who are immunocompromised, with potential pathogens emerging as keystone taxa within these networks. Additionally, differential gene expression between immunocompromised and non-immunocompromised patients was evident, particularly in the downregulation of immune-related pathways. The relationship between the microbiome, host genetic expression, and clinical indicators is complex and multifaceted. To gain a deeper understanding of the pathophysiological mechanisms underlying immunocompromise in sepsis, it is imperative to further elucidate the intricate interactions between host immune responses and microbial dynamics.

## MATERIALS AND METHODS

### Patient enrollment

A retrospective analysis was conducted on patients with sepsis-induced ALI admitted to the ICU of the First Affiliated Hospital of Jinan University between February 2019 and August 2021. The inclusion criteria were as follows: (i) age over 18 years; (ii) admission with suspected pulmonary infection and a sequential organ failure assessment (SOFA) score of ≥2; (iii) collection of bronchoalveolar lavage fluid during hospitalization for metagenomic next-generation sequencing (mNGS) analysis. In this study, the definition of pulmonary infection was based on the diagnosis made by the clinical team during the hospital stay. This diagnosis incorporated the patient’s clinical presentation, imaging examination (such as chest X-rays or CT scans), and laboratory test results. Additionally, when retrospectively including these cases, a physician re-evaluated the diagnosis. The exclusion criteria included: (i) ALI caused by non-infectious factors and (ii) incomplete clinical data.

We recruited 79 patients at the beginning. Due to incomplete clinical data or non-compliance with inclusion criteria, a total of 54 patients were enrolled in the study. Based on their immunological status, participants were divided into two groups: the immunocompromised host (ICH) group and the control group (non-immunocompromised). According to previous research (50, 51), immunosuppression was defined as meeting any of the following conditions: (i) HIV infection with a CD4^+^ T^−^ lymphocyte count <200 cells/µL; (ii) consumption of 20 mg of glucocorticoids daily for more than 14 days or use of immunosuppressants, biological immunomodulators, and anti-rheumatic drugs; (iii) malignant hematological diseases; (iv) hematopoietic stem cell or solid organ transplantation; (v) recent treatment of solid tumors with chemotherapeutic agents; (vi) primary immunodeficiency disease. Ultimately, there were 18 patients in the ICH group and 36 in the control group. Among the 18 patients in ICH group, 7 received prednisone, 10 received chemotherapy, and 1 had malignant hematological diseases.

### Clinical data and sample collection

Clinical data were collected and analyzed by two resident physicians. The patients’ demographic characteristics, medical history, laboratory test results, clinical information, treatment, and prognosis were extracted from the medical record system. These data were subsequently reviewed by a senior physician.

Following the recommended procedure by the American Thoracic Society, all patients underwent BALF collection under local anesthesia via fiberoptic bronchoscopy (52). All samples were collected in sterile containers and transported at low temperature to Guangzhou Vision Medicals Co., Ltd. for metagenomic sequencing.

### Metagenomic sequencing

DNA was extracted from all samples using a QIAamp UCP Pathogen DNA Kit (Qiagen) following the manufacturer’s instructions. Human DNA was removed using Benzonase (Qiagen) and Tween20 (Sigma). Total RNA was extracted with a QIAamp Viral RNA Kit (Qiagen) and ribosomal RNA was removed by a Ribo-Zero rRNA Removal Kit (Illumina). cDNA was generated using reverse transcriptase and dNTPs (Thermo Fisher). Libraries were constructed for the DNA and cDNA samples using a Nextera XT DNA Library Prep Kit (Illumina, San Diego, CA). Library was quality assessed by Qubit dsDNA HS Assay kit followed by High Sensitivity DNA kit (Agilent) on an Agilent 2100 Bioanalyzer. Library pools were then loaded onto an Illumina Nextseq 550Dx sequencer for 75 cycles of single-end sequencing to generate approximately 20 million reads for each library. For negative controls, we also prepared PBMC samples with 10^5^ cells/mL from healthy donors in parallel with each batch, using the same protocol, and sterile deionized water was extracted alongside the specimens to serve as non-template controls (NTC).

### Bioinformatics analyses

Trimmomatic was used to remove low-quality reads, adapter contamination, and duplicate reads, as well as those shorter than 50 bp. Low complexity reads were removed by Kcomplexity with default parameters. Human sequence data were identified and excluded by mapping to a human reference genome (hg38) using Burrows-Wheeler Aligner software. We designed a set of criteria similar to the National Center for Biotechnology Information (NCBI) criteria for selecting representative assembly for microorganisms (bacteria, viruses, fungi, protozoa, and other multicellular eukaryotic pathogens) from the NCBI Nucleotide and Genome databases. Pathogen lists were selected according to three references: (i) Johns Hopkins ABX Guide (https://www.hopkinsguides.com/hopkins/index/Johns_Hopkins_ABX_Guide/Pathogens), (ii) Manual of Clinical Microbiology (https://www.clinmicronow.org/doi/book/10.1128/9781683670438.MCM), and (iii) clinical case reports or research articles published in current peer-reviewed journals (53). The final database consisted of about 18,562 genomes. Microbial reads were aligned to database with SNAP v1.0beta.18. Virus-positive detection results (DNA or RNA viruses) were defined as the coverage of three or more non-overlapping regions on the genome. A positive detection was reported for a given species or genus if the reads per million (RPM) ratio, or RPM-r was ≥5, where the RPM-r was defined as the RPMsample/RPMNC (i.e., the RPM corresponding to a given species or genus in the clinical sample divided by the RPM in the NC/negative control). In addition, to minimize cross-species misalignments among closely related microorganisms, we penalized (reduced) the RPM of microorganisms sharing a genus or family designation if the species or genus appeared in non-template controls. A penalty of 5% was used for species.

### Transcriptomic sequencing

RNA extraction was performed using the QIAamp Viral RNA Kit from QIAGEN, and ribosomal RNA depletion was carried out using the Illumina Ribo-Zero rRNA Removal Kit. For cDNA synthesis, the reverse transcription enzyme and dNTPs from Thermo Fisher were used. BALF RNA was extracted using the QIAamp Circulating Nucleic Acid Kit from QIAGEN, and all procedures were conducted following the official protocols provided by the respective manufacturers. To construct cDNA libraries, the Illumina Nextera XT DNA Library Prep Kit was employed. Library quality was assessed using the AGILENT Qubit dsDNA HS Assay kit, and the insert size was confirmed to be as expected. Quantification of library concentration was achieved using Q-PCR to ensure library quality. Once the libraries passed quality control, they were sequenced on an Illumina NextSeq 550 platform. Different libraries were sequenced based on their effective concentration and the desired data output.

### Microbial analysis

Alpha diversity is employed to assess the diversity within samples, while beta diversity is used to compare the microbial community compositions between samples. The Simpson index and Shannon index were computed to estimate alpha diversity. Higher Shannon and Simpson indices indicate greater diversity within the community. Beta diversity was demonstrated using principal component analysis (PCA) based on the Bray-Curtis distance. To evaluate the lung microbiota network characteristics of two groups, we calculated Spearman coefficients for the top 100 species by relative abundance. Networks were constructed for species with Spearman correlation coefficients with absolute values greater than 0.6 and *P* values less than 0.05. Visualization and degree calculations of the network were performed using Cytoscape software (Version 3.7.1). Random forest model was constructed to identify microorganisms that most effectively distinguish between two groups. The model’s performance was evaluated using a 10-fold cross-validation method.

### Transcriptome analysis

Differential gene expression analysis was conducted using the DESeq2 package in RStudio software (54). The selection of differentially expressed genes (DEGs) was primarily based on fold change and adjusted *P* values as key metrics. In this study, the parameters for selecting DEGs were |log2 Fold Change| ≥1 and adjusted *P* < 0.05. The clusterProfiler package was utilized for Gene Ontology (GO) enrichment analysis and Kyoto Encyclopedia of Genes and Genomes (KEGG) pathway enrichment analysis of these genes (55). Additionally, we analyzed the expression of immune-related genes based on the gene list provided by the ImmPortDB database (56). The expression of interferon-induced genes was analyzed based on the gene list provided by Wu et al. in their study published in cell (57). The Wilcoxon rank-sum test was applied to evaluate significant differences in gene expression between different groups of samples.

### Statistical analysis

Quantitative data that follows a normal distribution or closely approximates it is presented as mean ± standard deviation and is analyzed using the *t*-test. Quantitative data that exhibits skewness is expressed as median (percentiles: P25, P75) and analyzed using the Wilcoxon rank sum test. Categorical data is represented as frequencies and analyzed using the chi-square test.

## Data Availability

Raw sequencing data have been publicly deposited and are available at the NCBI Sequence Read Archive, with BioProject accession no. PRJNA1107343.

## AFTER EPUB

This article was published on 18 October 2024 with an error in the equal contribution note. The note was corrected in the current version, posted on 13 November 2024.

## SUPPLEMENTAL MATERIAL

Supplemental figures spectrum.01424-24-s0001.pdf Fig. S1 and S2.

ASM does not own the copyrights to Supplemental Material that may be linked to, or accessed through, an article. The authors have granted ASM a non-exclusive, world-wide license to publish the Supplemental Material files. Please contact the corresponding author directly for reuse.
