# Supplementary material for: Multi-omics analysis reveals the interplay between pulmonary microbiome and host in immunocompromised patients with sepsis-induced acute lung injury
Source: Microbiol Spectr. 2024 Oct 18;12(12):e01424-24. doi: 10.1128/spectrum.01424-24 (PMC11619524; doi:10.1128/spectrum.01424-24)
Supplement: Supplemental figures — Fig. S1 and S2. [file spectrum.01424-24-s0001.pdf]

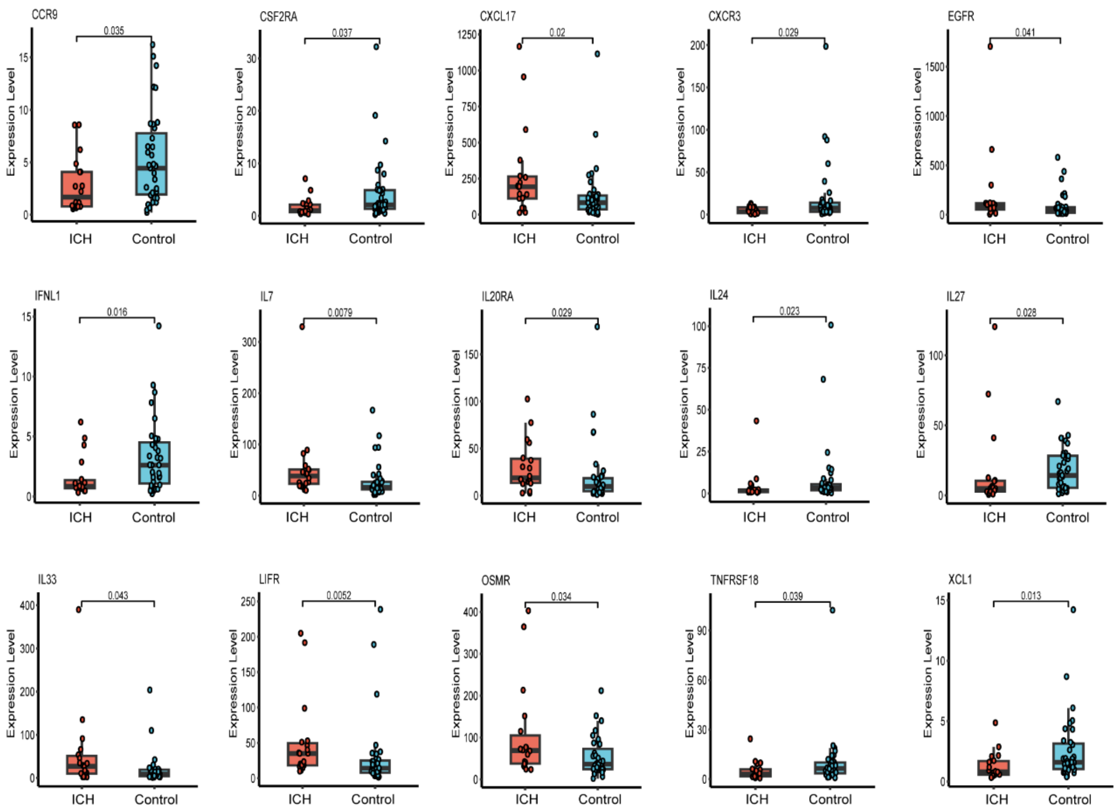

Supplemental Figure S1 Differential immune-related genes between ICH and control groups.

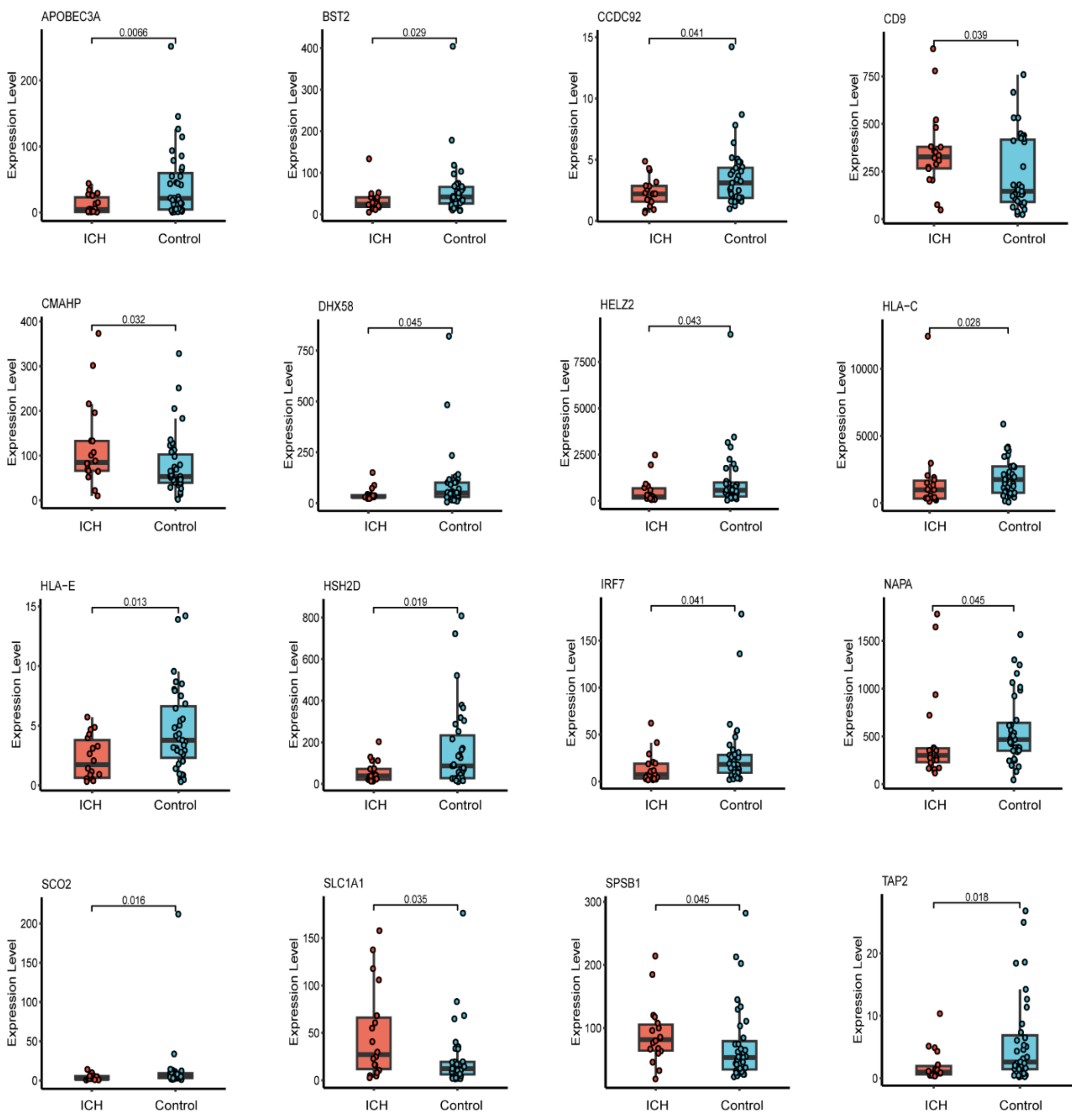

Supplemental Figure S2 Differential interferon-induced genes between ICH and control groups.
